# Whole-transcriptome profiling reveals potential biomarkers for the reversal of thymic epithelial cell senescence by umbilical cord mesenchymal stem cells

**DOI:** 10.18632/aging.205738

**Published:** 2024-04-17

**Authors:** Zai-Ling Yang, Chuan Tian, Jie He, Hang Pan, Guang-Ping Ruan, Jing Zhao, Kai Wang, Xing-Hua Pan, Xiang-Qing Zhu

**Affiliations:** 1 The Basic Medical Laboratory of the 920th Hospital of Joint Logistics Support Force of PLA, The Transfer Medicine Key Laboratory of Cell Therapy Technology of Yunan, The Integrated Engineering Laboratory of Cell Biological Medicine of State and Regions, Kunming 650032, Yunnan, China; 2 The Second People’s Hospital of Guiyang, Medical Laboratory, Guiyang 550023, Guizhou, China

**Keywords:** TECs senescence, UCMSCs, ceRNA, RNA-seq

## Abstract

Background: Reduced numbers and dysfunction of thymic epithelial cells (TECs) are important factors of thymic degeneration. Previous studies have found that umbilical cord mesenchymal stem cells (UCMSCs) reverse the structure and function of the senescent thymus *in vivo*. However, the transcriptomic regulation mechanism is unclear.

Methods: TECs were cultured with H_2_O_2_ for 72 hours to induce senescence. UCMSCs were cocultured with senescent TECs for 48 hours to detect SA-β-gal, P16 and Ki67. The cocultured TECs were collected for lncRNA, mRNA and miRNA sequencing to establish a competitive endogenous regulatory network (ceRNA). And RT-qPCR, immunofluorescence staining, and western blot were used to identified key genes.

Results: Our results showed that H_2_O_2_ induced TEC aging and that UCMSCs reversed these changes. Compared with those in aged TECs, 2260 DE mRNAs, 1033 DE lncRNAs and 67 DE miRNAs were differentially expressed, and these changes were reversed by coculturing the cells with UCMSCs. Differential mRNA enrichment analysis of ceRNA regulation revealed that the PI3K-AKT pathway was a significant signaling pathway. UCMSC coculture upregulated VEGFA, which is the upstream factor of the PI3K-AKT signaling pathway, and the expression of the key proteins PI3K and AKT. Thus, the expression of the cell cycle suppressor P27, which is downstream of the PI3K-AKT signaling pathway, was downregulated, while the expression of the cell cycle regulators CDK2 and CCNE was upregulated.

Conclusion: UCMSC coculture upregulated the expression of VEGFA, activated the PI3K-AKT signaling pathway, increased the expression of CDK2 and CCNE, decreased the expression of P27, and promoted the proliferation of TECs.

## INTRODUCTION

Thymic epithelial cells (TECs), the major constituent of thymic stromal cells, are crucial for the thymic milieu and all phases of T-cell maturation. The thymus is the principal immunological organ that produces both immune-competent and self-tolerant T cells [1–3]. Nonetheless, during the first several months of life, the thymus progressively atrophies, which causes an ongoing decrease in thymic cell structure and disruption of the thymic stromal milieu. This includes the disappearance of discrete cortex-medullary junctions and a decrease in cortical and medullary TECs [4]. Due to the importance of the thymus gland, certain therapies, such as growth hormone, insulin growth factor (IGF), and keratinocyte growth factor (KGF) therapies, have been proposed to increase thymus production; however, these therapies are systemic and have unwanted side effects [5].

Neonatals’ umbilical cord tissue is where adult stem cells known as umbilical cord mesenchymal stem cells (UCMSCs) are extracted. Their biological characteristics, such as their capacity for multidirectional differentiation, are comparable to mesenchymal stem cells obtained from bone marrow, fat tissue, and pulp of tooth [6]. Exogenous UCMSCs can exert antioxidative stress by secreting cytokines, inhibiting apoptosis, promoting autophagy, and improving the viability of tissue cells [7–9]. Recent studies have shown that UCMSCs upregulate aging genes and those linked to autophagy and oxidative stress, preserving the integrity and function of the thymus in old C57BL/6J mice [10]. Moreover, following UCMSC therapy, transcriptomic differences in the thymus tissue of aged macaques occur [11]. However, the mechanism by which UCMSCs reverse thymic aging remains poorly understood.

MicroRNAs, also known as short noncoding RNAs, are a type of RNA that, by blocking mRNA, regulate several of numerous processes in biology, notably cell growth, differentiation, and death [12]. Previous RNA-seq-based studies have found that miRNAs can be used as good biological indicators for studying aging and age-related diseases [13]. Noncoding RNA transcripts larger than 200 nucleotides are known as long noncoding RNAs (lncRNAs), and they play a role in cell proliferation, transcriptional regulation, and the growth and aging of many tissues and organs [14]. As molecular sponges, lncRNAs compete with miRNAs either directly or indirectly, ultimately lessening their impact on mRNAs. This creates a competitive network of endogenous RNAs, or ceRNAs. The lncRNA-related ceRNA network has been associated with the pathogenesis of aging-related diseases [15, 16]. Therefore, by analyzing the ceRNA network, we were able to study the mechanism by which UCMSCs reverse thymic aging.

Using an H_2_O_2_-induced TEC senescence model, we evaluated the impact of UCMSCs on reversing senescence in the current research. Additionally, in order to identify changes in mRNA, miRNA, and lncRNA profiles and build ceRNA networks connected to UCMSCs reversing TEC senescence, we used transcriptome analysis based on RNA sequencing.

## RESULTS

**Figure 1:**
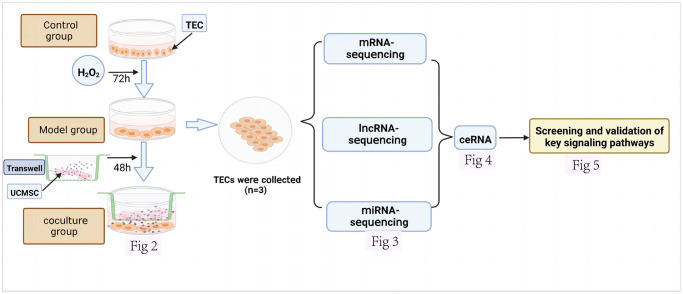
**Design of an experimental investigation.** The various datasets produced are displayed together with the corresponding figure numbers.

**Figure 2:**
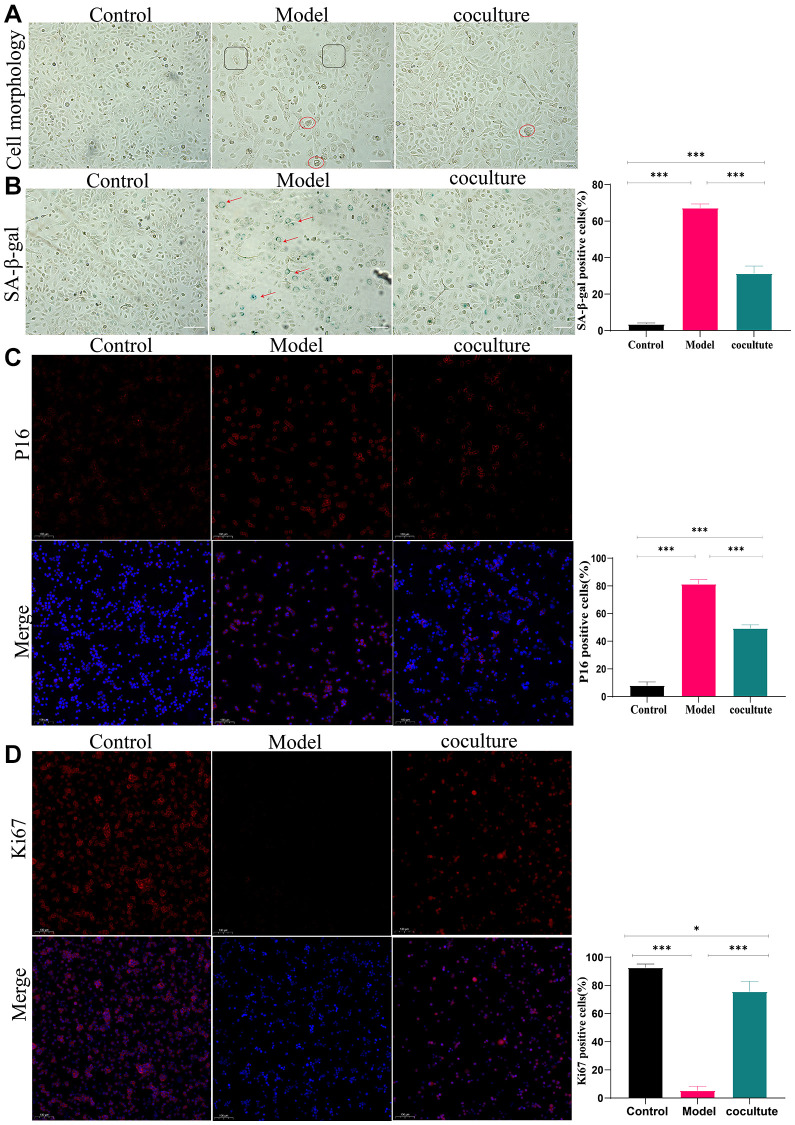
**Interference effect of UCMSCs on senescent TECs *in vitro*.** (**A**) The morphology of TECs (100×). (**B**) The activity of β-galactosidase was determined using β-galactosidase staining (100×). (**C**) P16 expression was detected by immunocytochemical staining (100×). (**D**) Ki67 staining was performed to observe TEC proliferation (100×) (the measured values represent the mean ± standard deviation (X ± s)) of three replicate experiments. ^*^P < 0.05, ^**^P < 0.01, ^***^P < 0.001).

**Figure 3:**
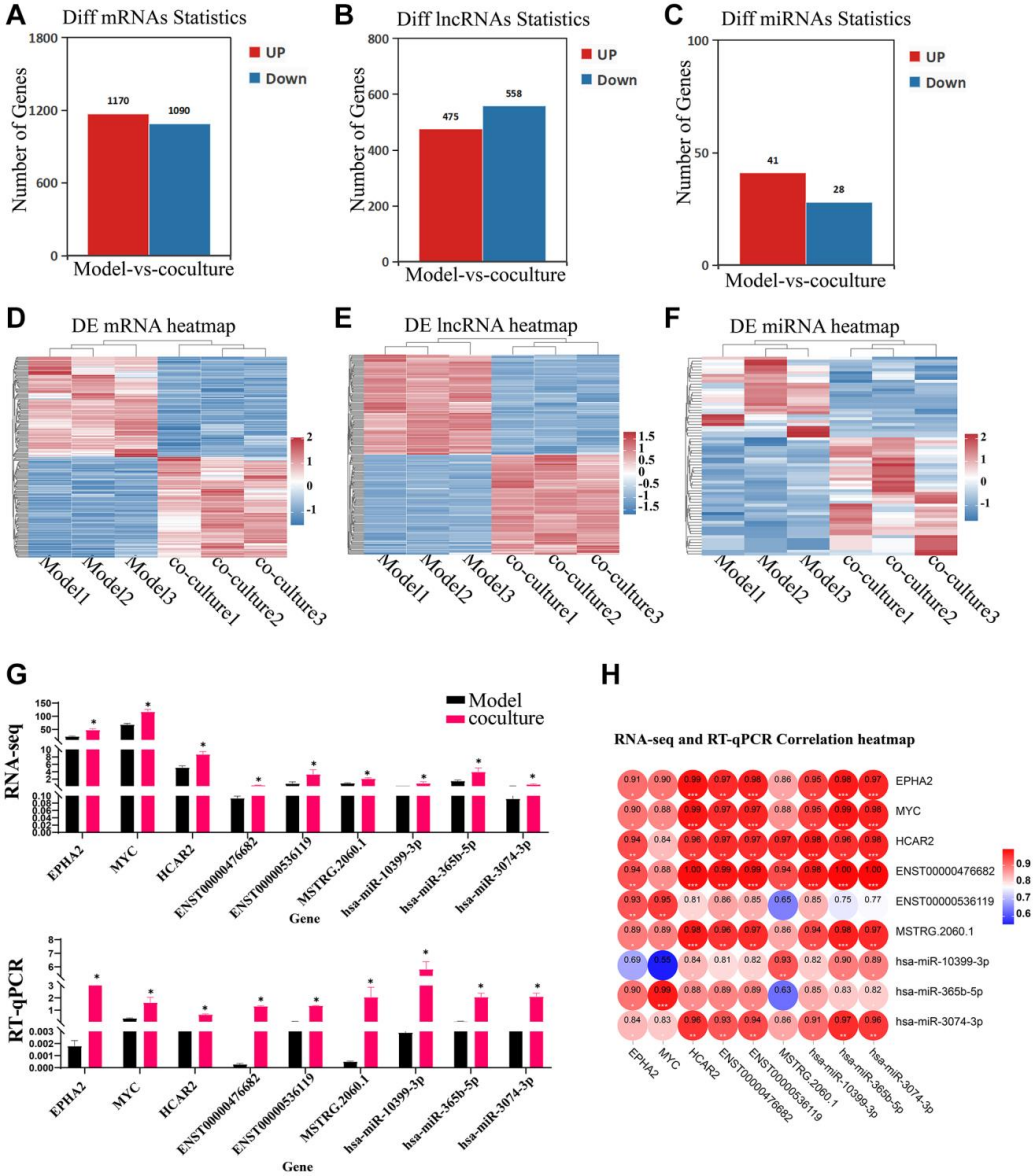
**Identification of DEGs, DELs and DEMs.** (**A**–**C**) Bar chart of DE mRNAs, lncRNAs and miRNAs. (**D**–**F**) Heatmap of DE mRNAs, lncRNAs and miRNAs. (**G**) RT-qPCR validating of the RNA sequencing data using 3 DE mRNAs, lncRNAs and DE miRNAs chosen at random (the upper panel represents the fpkm of RNA-seq, and the following graph represents the expression of RT-qPCR). (**H**) RNA-seq and RT-qPCR correlation heatmap. The measured values represent the mean ± standard deviation (X ± s) of three replicate experiments. ^*^P < 0.05.

**Figure 4:**
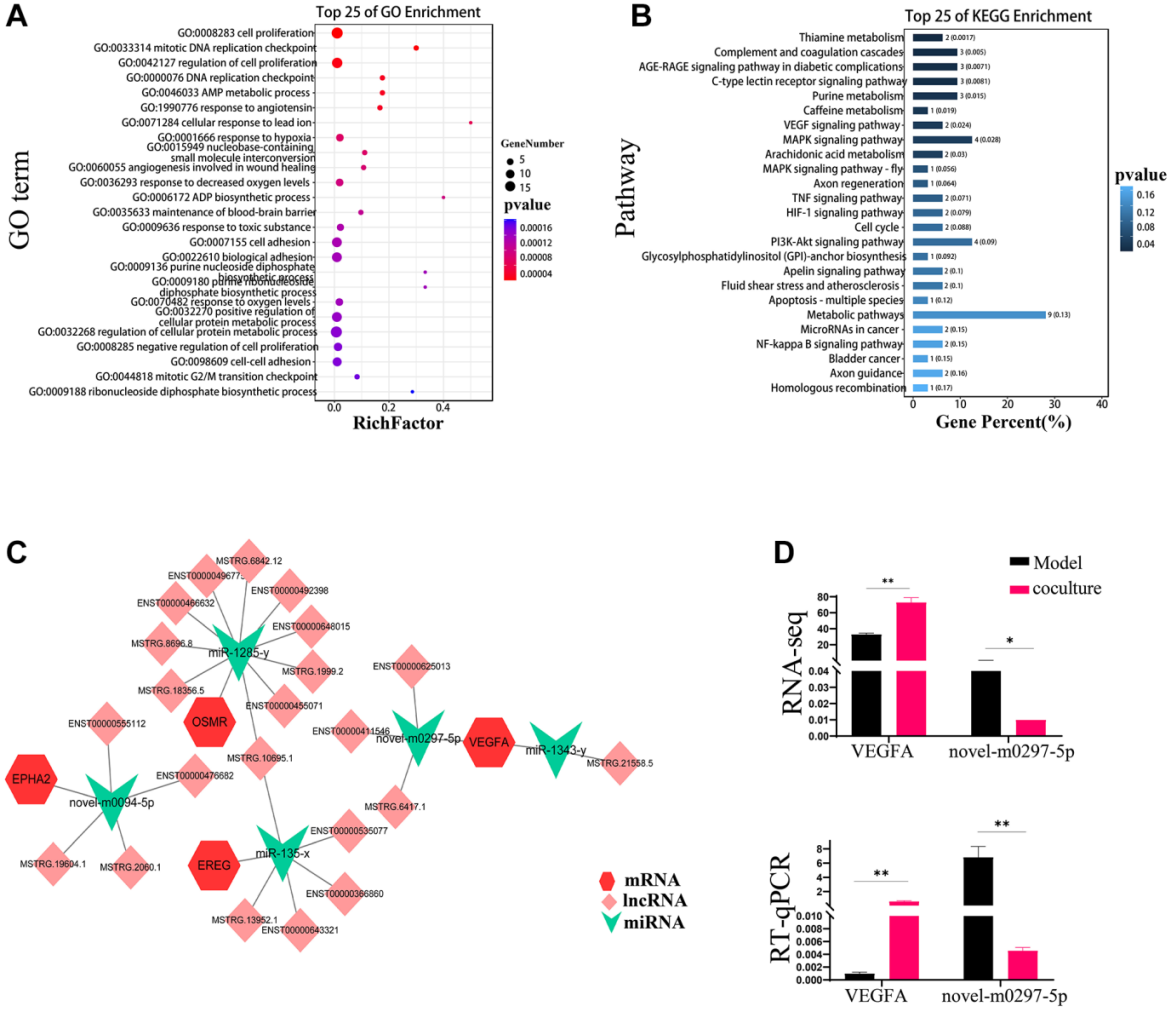
**ceRNAs reverse TEC senescence via UCMSCs.** (**A**) GO enrichment analysis inside the ceRNA network for mRNAs. (**B**) KEGG enrichment analysis of mRNAs in ceRNA networks. (**C**) Distinct mRNA, miRNA, and lncRNA expression patterns within the ceRNA network. miRNAs are represented by the green inverted triangle nodes, lncRNAs by the light pink quadrilateral nodes, and mRNAs by the red hexagon nodes. (**D**) Objective results of VEGFA and novel-m0297-5p RNA sequencing were verified by RT-PCR. The measured values represent the mean ± standard deviation (X ± s) of three replicate experiments. ^*^P < 0.05, ^**^P < 0.01.

**Figure 5:**
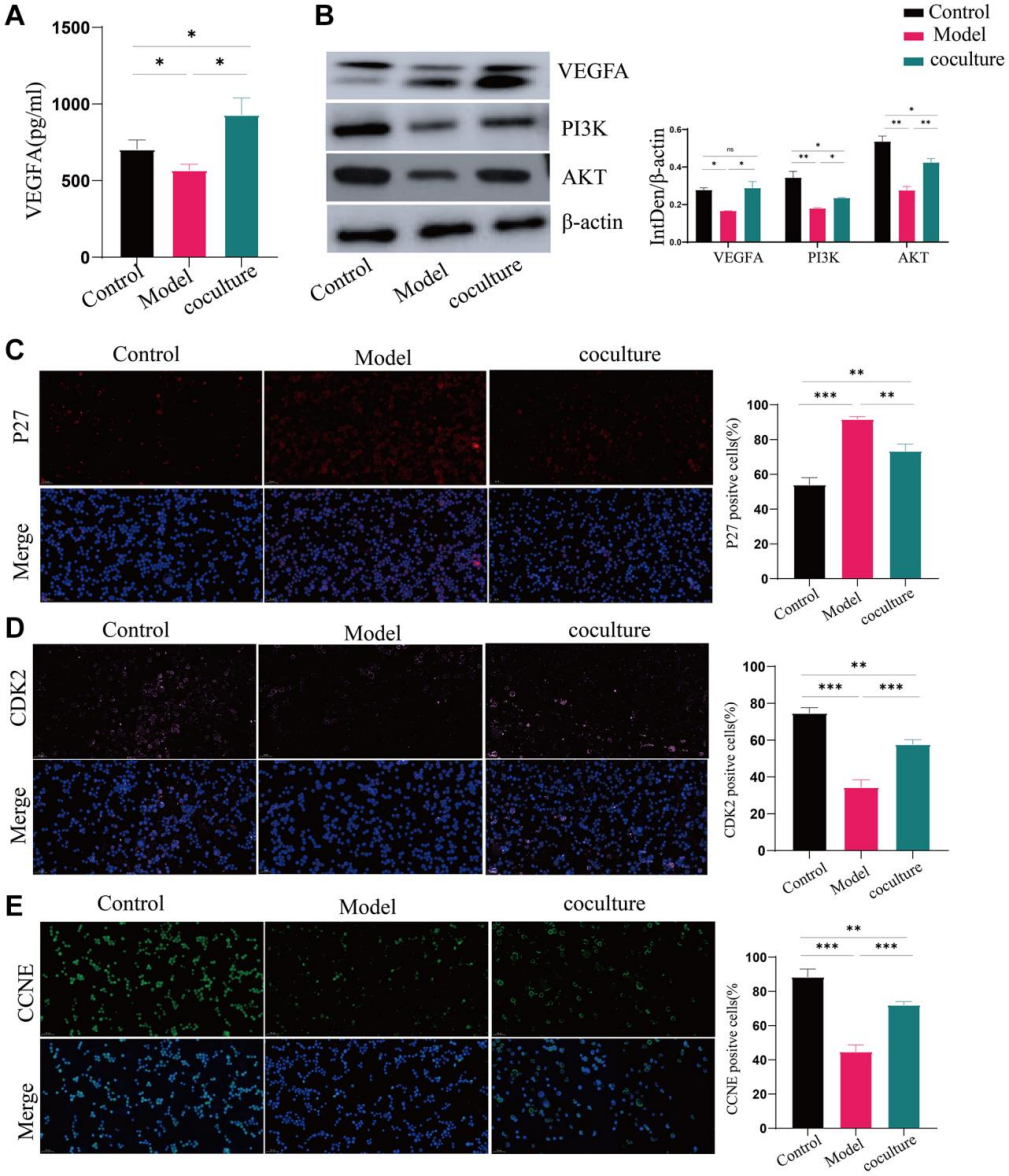
**UCMSCs boost the activity of the PI3K-AKT pathway in senescent TECs.** (**A**) VEGFA expression was detected by ELISA. (**B**) The PI3K-AKT signaling pathway proteins VEGFA, PI3K, and AKT were shown to be expressed by western blotting, with β-actin serving as a control. (**C**–**E**) After the PI3K-AKT signaling pathway, immunofluorescence was used to measure the levels of p27, CDK2, and CCNE, which are important for cell cycle regulation (100×). The measured values represent the mean ± standard deviation (X ± s) of three replicate experiments. ^*^P < 0.05, ^**^P < 0.01, ^***^P < 0.001).

Figure 1 depicts the experiment’s whole design process. The senescence model was established by culturing normal TECs with H_2_O_2_ for 72 h. Then, after 48 hours of coculture between UCMSCs and senescent TECs, the coculture system was evaluated. (Figure 2). These cells were used as experimental sources for mRNA, miRNA, and lncRNA sequencing and Functional enrichment analysis (Figure 3). ceRNA regulatory networks related to key signaling pathways were established (Figure 4) and functional validation (Figure 5).

### Interference effect of UCMSCs on senescent TECs *in vitro*

In our study, TECs were cocultured with H_2_O_2_ for 72 hours, followed by treatment with UCMSCs for 48 hours. Under a fluorescence inverted microscope, in contrast to those in the control group, the TECs in the model group were initially fat and wide, with a low number of cells and an increased amount of intracellular vesicles. The formation of TECs improved, their number increased, and their intracellular vacuoles decreased following coculture with UCMSCs (Figure 2A). Furthermore, SA-β-galactosidase staining (increased activity of β-galactosidase makes senescent cells look blue.) In the control group, 3.56 ± 0.66% of the TECs were stained blue, but in the model group, 67.33 ± 2.08%, and in the coculture group, 31.33 ± 4.05% were stained blue (Figure 2B). Subsequently, the expression of the P16 protein was determined by immunocytochemical staining, and the outcomes revealed that in the control group, 8 ± 2.64% of the cells expressed the P16 protein, but in the model group and coculture group, the percentages were 81.33 ± 3.21% and 49.33 ± 2.51%, respectively (Figure 2C). Finally, Ki67 is a cytonuclear protein, which is also a frequently used marker of cell proliferation, and senescent cells are marked by permanent exit from the cell cycle, so senescent cells do not express Ki67. Immunocell staining showed that Ki67 levels were 92.66 ± 2.51% in the control group, 5.33 ± 3.05% in the model group, and 75.66 ± 7.23% in the co-culture group (Figure 2D). These results indicate that TECs begin to age after treatment with 200 μm H_2_O_2_ for 72 h, and UCMSCs reverse these changes in aging TECs.

### Transcriptome alterations in senescent TECs following coculture with UCMSCs

To understand the effects of UCMSC on mRNA, lncRNAs and miRNA expression in senescent TEC, Illumina HiSeq^™^ 4000 was used to detect TEC in Model group and UCMSC co-culture groups. The differential expression thresholds were a fold change ≥1.2 and *p*-value < 0.05. Among these, 2260 DE mRNAs (1,170 upregulated mRNAs and 1,090 downregulated mRNAs), 1033 DE lncRNAs (475 upregulated lncRNAs and 558 downregulated lncRNAs) and 67 DE miRNAs (41 upregulated miRNAs and 28 downregulated miRNAs) were detected. To analyze the amounts of DE mRNAs, lncRNAs, and DE miRNAs expression, a bar chart was created. (Figure 3A–3C). To demonstrate variations in DE mRNA lncRNAs and DE miRNA expression structure samples, hierarchical clustering was used (Figure 3D–3F). Three DEGs, three DEMs, and three DELs were chosen at random for RT-qPCR analysis to confirm the accuracy of our whole-transcriptome data (Figure 3G, 3H). The repeatability and dependability of the data were demonstrated by the fact that the fluctuations in the transcriptome data matched those of the RT-qPCR data, with correlations better than 0.8. These results suggest that UCMSC coculture induces transcriptomic differences.

### ceRNAs that reverse TEC senescence via UCMSCs

To analyze the role of these different RNAs in reversing UCMSC-related TEC senescence, we established a ceRNA regulatory network consisting of DEmiRNAs, DElncRNAs, and DEmRNAs and used TargetScan scores >90 genes (Supplementary Table 1). To reveal the possible roles of the screened DE mRNAs, we carried out GO and KEGG analyses of pathways. The results showed that the GO terms “cell proliferation” and “cell cycle” were considerably more enriched in the UCMSC coculture group than in the model group (Figure 4A). The PI3K-AKT signaling pathway was among the 25 pathways with KEGG enrichment, as shown in Figure 4B. Along with regulating cell division, the cell cycle, and other critical biological activities, the PI3K-AKT signaling system is tightly linked to the aging pathway. Therefore, we focused on the PI3K-AKT signaling pathway in our subsequent analysis. We used Cytoscape_v3.7.2 software to construct and visualize lncRNA-miRNA–mRNA ceRNA networks enriched in the PI3K–AKT signaling pathway (Figure 4C) This network included 4 mRNAs, 5 miRNAs, and 21 lncRNAs, and intricate relationships were present among these ceRNAs. VEGFA is an upstream molecule of PI3K-AKT that regulates the activation and inactivation of signaling pathways. Furthermore, VEGFA was expected to be a target gene of novel-m0297-5p in the ceRNA network. Our RT-qPCR results also confirmed these findings (Figure 4D).

### UCMSCs reverse TEC senescence by activating PI3K-AKT pathway through up-regulation of VEGFA

VEGFA expression was dramatically upregulated in UCMSC-cocultured cells, according to the ELISA data, and much reduced in the model group compared to the control group (Figure 5A). Using western blot analysis, the expression of the vital nodal proteins VEGFA, PI3K, and AKT in the PI3K-AKT signaling pathway was also examined. VEGFA, PI3K, and AKT protein levels were substantially diminished in the model group in comparison with the control group. The expression levels of these genes markedly increased when the cells were cocultured with UCMSCs (Figure 5B). These results suggest that UCMSCs promote the PI3K-AKT pathway by upregulating VEGFA. We also measured the expression of the downstream regulatory factors p27, CDK2 and CCNE in the PI3K-AKT pathway. Figure 5C–5E demonstrates that whereas p27 expression levels were considerably greater in the model group compared to in the control group, CDK2 and CCNE expression levels were considerably lower in the model group. The expression of CDK2 and CCNE significantly grew and p27 substantially reduced in the cells after they were cocultured with UCMSCs. These findings demonstrated that UCMSCs activated the PI3K-AKT pathway through VEGFA overexpression, hence reversing TEC senescence.

## DISCUSSION

Because of their high self-renewal capacity, capacity to fight oxidative stress and inflammation, and capacity to release a variety of cytokines, UCMSCs have been suggested to be crucial for reversing thymic aging [17]. In addition, TECs are fundamental to the three-dimensional structure of the thymus and are crucial for organ growth [18, 19]. In this study, we treated normal TECs with H_2_O_2_ to simulate *in vivo* thymic senescence. As an oxidant of tiny molecules, H_2_O_2_ readily causes cell senescence through oxidative stress to the cell membrane and represents a fast, convenient and reliable tool for establishing senescent cell models [20]. To investigate the interaction mechanism, we cocultured aged TECs with UCMSCs in a Transwell system. Remarkably, the cellular senescence morphology improved, β-galactosidase and P16 levels decreased, and Ki67 expression increased after the coculture of senescent TECs with UCMSCs, indicating that UCMSCs restored the levels of these senescence-related factors [21–24]. Furthermore, an aging TEC model was effectively created, and the aging of TECs was reversed by UCMSCs. To elucidate the mechanism by which UCMSCs reverse senescence, we performed a series of high-throughput sequencing experiments.

Through high-throughput sequencing, we identified 2260 DE mRNAs, 1033 DE lncRNAs, and 67 DE miRNAs that could be connected to the ability of UCMSCs to prevent TEC senescence. Three DELs, three DEMs, and three DEGs were randomly selected for RT-qPCR verification to assess the precision of the whole-transcriptome data. The results validated the excellent dependability of our data (R2 >0.8) and revealed a similar pattern across the two studies. VEGFA may be a possible biomarker for reversing TEC senescence in UCMSCs according to our ceRNA-targeted regulation and KEGG enrichment studies. A constituent of the VEGF family, VEGFA is essential for the proliferation and angiogenesis of vascular endothelial cells and can supply tissues or cells with nutrients to prevent aging of the cells for a variety of reasons [25, 26]. It has been suggested that VEGFA is a potential novel therapy for neurodegenerative illnesses. A study of graduate students showed that the cognitive performance of a twofold transgenic AD mouse model may improve, and high VEGFA levels may be maintained by transplanting mesenchymal stem cells [27]. It is speculated that MSCs improve cell damage mainly through paracrine mechanisms, and vascular endothelial growth factor (VEGF) and hepatocyte growth factor (HGF) secreted by MSCs are two essential factors that promote cell proliferation [28]. Our study also confirmed the increased expression of VEGFA in the supernatant of UCMSC-cocultured TECs. Therefore, we hypothesized that UCMSCs reverse TEC senescence by secreting VEGFA and acting on senescent TECs. VEGFA attaches to and activates cell membrane receptors as a cytokine. This signal reaches the PI3K/AKT pathway, where it is activated together with downstream components. According to our research, the expression of PI3K, a crucial protein in the PI3K-AKT signaling pathway, and AKT increased during UCMSC coculture, indicating that VEGFA can upregulate the PI3K-AKT signaling pathway.

The PI3K/AKT pathway is a prominent signal transduction pathway that is intimately linked to the cell cycle, survival, and proliferation. Through the phosphorylation of several cytoplasmic proteins during senescence, AKT, which is activated by PI3K, can support cell growth and survival. Development factors and insulin are only two examples of the numerous variables that can regulate AKT [29]. The downstream components of the PI3K/AKT signaling pathway, P27, CDK2, and CCNE, can control the cell cycle and proliferation [30, 31]. Among them, the P27 protein is an important regulatory protein in eukaryotes and is capable of inhibiting a variety of CDK-cyclin complexes; specifically, P27 inhibits CDK2 and cyclin a (CCNA) or E (CCNE) complexes and is essential for the negative control of cell growth [32, 33]. H_2_O_2_ treatment in our study was linked to increased production of P27, significant suppression of the PI3K/AKT signaling pathway, and reduced VEGFA expression in TECs. These effects may be reversed after. UCMSCs elevated VEGFA expression in senescent TECs and activated PI3K and Akt, and the activated PI3K-AKT pathway negatively regulated P27, subsequently abrogating the inhibitory effects of P27 on the Cdk2/cyclin A and cyclin E complexes. It promoted cell division and multiplication and increased kinase activity from the late G1 phase to the S phase.

However, the biological processes and mechanisms of novel-0297-5p and miR-1343-y are rarely reported. We constructed a ceRNA network related to the PI3K-AKT signaling pathway to reveal the mechanism by which UCMSCs reverse TEC senescence. Based on our hypothesis, novel-0297-5p and miR-1343-y may target VEGFA and alter the PI3K-AKT signaling pathway to control the cell cycle and proliferation, providing new information on how UCMSCs reverse TEC senescence.

## CONCLUSIONS

We induced senescence of TECs with H_2_O_2_* in vitro*, and by coculturing senescent TECs with UCMSCs, the levels of genes linked to senescence and the activity of antioxidant enzymes could be reduced, thereby reversing H_2_O_2_-induced TEC senescence. The reverse process of TEC aging through UCMSCs is associated with the establishment of a ceRNA network that regulates the production of VEGFA, activates the PI3K-AKT signaling pathway, and controls the expression of CDK2/CCNE and p27, two important cell cycle proteins.

## MATERIALS AND METHODS

### Cell source

UCMSCs were obtained from the Chinese People’s Liberation Army’s 920th Hospital’s Cell Biotherapy Center’s Stem Cell Bank.

Meisen Biological Co., Ltd., provided the TECs, which were cultured in culture flasks with DMEM/F12 supplemented with 10% fetal bovine serum. They were then incubated at 37°C in an environment containing 5% carbon dioxide.

### Experimental groups

After fourth-generation thymus epithelial cells were collected and adjusted to a concentration of 5 × 10^5^ L^−1^, 100 μL of the six well petri dishes were added to a petri dishes plate filled with DMEM/F12 supplemented with 10% fetal bovine serum. After reaching an approximately 80% confluence, the cells were incubated with 200 μmol/L H_2_O_2_ (China Nanguo Co., Ltd., China) for 72 hours, and the supernatant was removed. After the fourth generation of UCMSC were gathered and adjusted to a cell concentration of 5 × 10^5^ L^−1^, the Transwell chamber was filled with 100 μL of cell suspension. The Transwells were then placed on the model group’s 6-well plate and cultivated for 48 hours.

### SA-β-gal activity staining

Following collection, TECs were once again cleaned with PBS, processed according to the Solarbio G1580 kit instructions, and examined under a standard optical microscope (ZEN2011) to determine the quantity of blue-positive cells in each of three fields of vision.

### Immunocytofluorescence revealed the expression of linked genes

After being harvested, the cultivated thymus epithelial cells were treated with paraformaldehyde. After 100 μL of membrane-permeable working solution was added, the membrane was covered with 3% bovine serum albumin and incubated for 20 minutes. P16, Ki67, P27, CDK2, and CCNE antibodies were added, and the samples were incubated at 4°C for an additional 30 minutes after isolation. CY3/CY5/FITC-conjugated goat anti-rabbit antibodies were added, and the sections were incubated for 50 minutes, followed by 15 minutes of incubation with DAPI. Images were taken, and the sections were examined with a fluorescence microscope. (Light is emitted by DAPI in blue, CY3 in red, FITC in green, and CY5 in pink).

### RNA sequencing and data analysis

After thymic epithelial cells were collected, total RNA was extracted using Trizol (Invitrogen, USA). The NEBNext^®^Ultra^™^ Directional RNA Library Prep Kit for Illumina^®^ (NEB, USA) was utilized to prepare sequencing libraries, and the Agilent bioanalyzer system was used to evaluate the libraries’ quality. Following cluster creation, the library was sequenced using an Illumina HiSeq 4000 platform, yielding 150 bp paired-end reads. Clean reads were obtained by eliminating low-quality reads, adaptors, and poly-N from the raw data. Transcripts without coding potential were regarded as candidate sets for new lncRNAs, whereas transcripts with anticipated coding potential were removed.

### Analysis of differential expression and gene ontology enrichment

The DEseq2 program was employed to ascertain the differentially expressed mRNAs, lncRNAs, and miRNAs. In various comparisons, genes with a *P*-value < 0.05 and a log2FC > 2 were considered differentially expressed. The GO enrichment process for TopGO was carried out.

### Establishing the ceRNA network

Initially, we looked for negative correlations between the levels of their expression and targeting interactions between miRNAs and potential ceRNAs. Subsequently, we examined potential positive associations between the expression levels of potential ceRNAs. The degree to which the same miRNA was enriched was used to evaluate putative ceRNA binding. Cytoscape v3.01 was used to construct a lncRNA-miRNA-mRNA network.

### Related gene verification using RT-qPCR

Total RNA was obtained using TRIzol^™^ reagent for quantitative PCR analysis following the supplier’s instructions (Carlsbad Invitrogen Company, USA). cDNA was amplified using random primers and reverse transcriptase (Goldenstar^™^ RT6, China). TsingKe Biotech Co., China gave instructions for RT-qPCR, which was carried out using a CFX96^™^ Real-Time System (Applied 2720) and SYBR Green Master Mix. As internal controls, the expression levels of GAPDH and U6 were compared to the expression levels of lncRNAs, mRNAs, and miRNAs, and the 2^−ΔΔT^ approach was utilized to normalize the gene expression levels. Primer information is detailed in Table 1.

**Table 1:** Primers used for RT-qPCR validation of mRNAs, lncRNAs, and candidate miRNAs.

	**Forward primer and reverse primer**
GAPDH	F: GGAGTCCACTGGCGTCTTCA
	R: GTCATGAGTCCTTCCACGATACC
U6	F: CTCGCTTCGGCAGCACA
	R: AACGCTTCACGAATTTGCGT
VEGFA	F: ATGGCAGAAGGAGGAGGG
	R: CGATTGGATGGCAGTAGC
EPHA2	F: CTCGGCTGGCTCACACAC
	R: GCTCGGGGCACTTCTTGT
MYC	F: CTGGATTTTTTTCGGGTAGTG
	R: CCTGGATGATGATGTTTTTGA
HCAR2	F: AACTATGTGAGGCGTTGGGA
	R: GCAAGAGATGATGGCTGCTG
ENST00000476682	F: GTTTAGGAGTGAGAGTAGCGC
	R: TGAAGAATCAGAAGAGGGTTT
ENST00000536119	F: CCTGTGCCTTCTACCTGTTCT
	R: TCCATCTGGCTCTTCTCTGTC
MSTRG.2060.1	F: TCGCTTCCTTGAATCCCTCTG
	R: TGAATCGCCTTGAACGCACAT
hsa-miR-10399-3p	CTCTCGGACAAGCTGTAGGTC
hsa-miR-365b-5p	AGGGACTTTCAGGGGCAGCTGT
hsa-miR-3074-3p	GATATCAGCTCAGTAGGCACCG
novel-m0297-5p	TAACTGTTTCTGCTAATTACT

### ELISA analysis

The cell supernatant was collected and centrifuged for 15 minutes at 3000 rpm, and the contents of the supernatants were examined in accordance with the guidelines supplied by the VEGFA ELISA Kit (EK183).

### Western blot analysis

After being collected, the cells were lysed in RIPA buffer (Solarbio, China). Following their isolation using Tris-Gly and 10% gel electrophoresis, the total TEC proteins were transferred to nitrocellulose membranes. After 1.5 hours of incubation at room temperature with 5% skim milk, the cell membrane was incubated overnight with primary antibodies against VEGFA (1:1000, ab46154), PI3K (1:1000, ab183957), and AKT (1:1000, GB111114). And gray value analysis was performed using ImageJ software.

### Statistical analysis

The statistical program SPSS 21.0 was used to analyze the data, and the results are displayed as the mean ± standard deviation. A *t*-test was utilized to compare the two groups, and one-way analysis of variance (ANOVA) was utilized to determine the significance of differences between the three groups.

## Supplementary Materials

Supplementary Table 1
