# Supplementary material for: Whole-transcriptome profiling reveals potential biomarkers for the reversal of thymic epithelial cell senescence by umbilical cord mesenchymal stem cells
Source: Aging (Albany NY). 2024 Apr 17;16(8):7009–21. doi: 10.18632/aging.205738 (PMC11087093; doi:10.18632/aging.205738)
Supplement: Supplementary Table 1 [file aging-16-205738-s001.docx]

Supplementary Table 1. The ceRNA network was constructed from lncRNA-miRNA-mRNA.

| lncRNA_ID | mRNA_ID | corr | miRNA_ID(both) | hyper.test.p |
| --- | --- | --- | --- | --- |
| ENST00000380176 | ENSG00000090776 | 0.910895623 | miR-1285-y | 0.002421308 |
| ENST00000444059 | ENSG00000104368 | 0.976492388 | miR-1285-y | 0.004842615 |
| ENST00000444059 | ENSG00000160214 | 0.900479044 | miR-1285-y | 0.02409554 |
| ENST00000444059 | ENSG00000171241 | 0.997822435 | miR-1285-y | 0.004842615 |
| ENST00000444059 | ENSG00000258674 | 0.942476027 | miR-1285-y | 0.004842615 |
| ENST00000444059 | ENSG00000277161 | 0.973229809 | miR-1285-y | 0.004842615 |
| ENST00000455071 | ENSG00000090776 | 0.927376527 | miR-1285-y | 0.007263923 |
| ENST00000455071 | ENSG00000105141 | 0.974209587 | miR-1285-y | 0.03596785 |
| ENST00000455071 | ENSG00000117525 | 0.980517367 | miR-1285-y | 0.007263923 |
| ENST00000455071 | ENSG00000136492 | 0.90731502 | miR-1285-y | 0.03596785 |
| ENST00000455071 | ENSG00000145623 | 0.986782354 | miR-1285-y | 0.02884446 |
| ENST00000455071 | ENSG00000148344 | 0.951142524 | miR-1285-y | 0.007263923 |
| ENST00000455071 | ENSG00000160072 | 0.931379858 | miR-1285-y | 0.02884446 |
| ENST00000455071 | ENSG00000160214 | 0.905905787 | miR-1285-y | 0.03596785 |
| ENST00000455071 | ENSG00000173530 | 0.956854789 | miR-1285-y | 0.03596785 |
| ENST00000465124 | ENSG00000136933 | 0.903390946 | miR-1285-y | 0.01452785 |
| ENST00000465124 | ENSG00000186854 | 0.951819087 | miR-1285-y | 0.01452785 |
| ENST00000466632 | ENSG00000090776 | 0.935730035 | miR-1285-y | 0.002421308 |
| ENST00000466632 | ENSG00000105141 | 0.924723478 | miR-1285-y | 0.01210654 |
| ENST00000466632 | ENSG00000117525 | 0.916617561 | miR-1285-y | 0.002421308 |
| ENST00000466632 | ENSG00000145623 | 0.959778057 | miR-1285-y | 0.00968523 |
| ENST00000466632 | ENSG00000148344 | 0.908564508 | miR-1285-y | 0.002421308 |
| ENST00000466632 | ENSG00000160072 | 0.917478612 | miR-1285-y | 0.00968523 |
| ENST00000466632 | ENSG00000177374 | 0.957342513 | miR-1285-y | 0.01210654 |
| ENST00000492398 | ENSG00000090776 | 0.922025227 | miR-1285-y | 0.01694915 |
| ENST00000492398 | ENSG00000117525 | 0.93168726 | miR-1285-y | 0.01694915 |
| ENST00000492398 | ENSG00000145623 | 0.904282806 | miR-1285-y | 0.001457059 |
| ENST00000496775 | ENSG00000090776 | 0.957974392 | miR-1285-y | 0.007263923 |
| ENST00000496775 | ENSG00000105141 | 0.980670666 | miR-1285-y | 0.03596785 |
| ENST00000496775 | ENSG00000107937 | 0.917796956 | miR-1285-y | 0.007263923 |
| ENST00000496775 | ENSG00000117525 | 0.981464545 | miR-1285-y | 0.007263923 |
| ENST00000496775 | ENSG00000132286 | 0.903612671 | miR-1285-y | 0.01449258 |
| ENST00000496775 | ENSG00000145623 | 0.978492801 | miR-1285-y | 0.02884446 |
| ENST00000496775 | ENSG00000148344 | 0.937048408 | miR-1285-y | 0.007263923 |
| ENST00000496775 | ENSG00000160072 | 0.954654386 | miR-1285-y | 0.02884446 |
| ENST00000496775 | ENSG00000160214 | 0.921072469 | miR-1285-y | 0.03596785 |
| ENST00000496775 | ENSG00000173530 | 0.941055349 | miR-1285-y | 0.03596785 |
| ENST00000496775 | ENSG00000177374 | 0.912023515 | miR-1285-y | 0.03596785 |
| ENST00000541754 | ENSG00000101945 | 0.92467098 | miR-1285-y | 0.00968523 |
| ENST00000541754 | ENSG00000117525 | 0.930176532 | miR-1285-y | 0.002421308 |
| ENST00000541754 | ENSG00000136492 | 0.910310412 | miR-1285-y | 0.01210654 |
| ENST00000541754 | ENSG00000146858 | 0.937628908 | miR-1285-y | 0.002421308 |
| ENST00000541754 | ENSG00000148344 | 0.915385885 | miR-1285-y | 0.002421308 |
| ENST00000541754 | ENSG00000165688 | 0.944111451 | miR-1285-y | 0.00968523 |
| ENST00000541754 | ENSG00000186854 | 0.960706815 | miR-1285-y | 0.002421308 |
| ENST00000541754 | ENSG00000267041 | 0.90903289 | miR-1285-y | 0.004842615 |
| ENST00000592456 | ENSG00000107937 | 0.927182026 | miR-1285-y | 0.00968523 |
| ENST00000648015 | ENSG00000090776 | 0.940064046 | miR-1285-y | 0.007263923 |
| ENST00000648015 | ENSG00000094804 | 0.90477674 | miR-1285-y | 0.02168607 |
| ENST00000648015 | ENSG00000105141 | 0.954519466 | miR-1285-y | 0.03596785 |
| ENST00000648015 | ENSG00000117525 | 0.979926396 | miR-1285-y | 0.007263923 |
| ENST00000648015 | ENSG00000136492 | 0.924054209 | miR-1285-y | 0.03596785 |
| ENST00000648015 | ENSG00000145623 | 0.947007319 | miR-1285-y | 0.02884446 |
| ENST00000648015 | ENSG00000148344 | 0.945689545 | miR-1285-y | 0.007263923 |
| ENST00000648015 | ENSG00000160214 | 0.913683579 | miR-1285-y | 0.03596785 |
| ENST00000648015 | ENSG00000165688 | 0.927044732 | miR-1285-y | 0.02884446 |
| ENST00000648015 | ENSG00000173530 | 0.919483129 | miR-1285-y | 0.03596785 |
| ENST00000662103 | ENSG00000104368 | 0.9752304 | miR-1285-y | 0.004842615 |
| ENST00000662103 | ENSG00000160214 | 0.9016766 | miR-1285-y | 0.02409554 |
| ENST00000662103 | ENSG00000171241 | 0.998598101 | miR-1285-y | 0.004842615 |
| ENST00000662103 | ENSG00000258674 | 0.943498959 | miR-1285-y | 0.004842615 |
| ENST00000662103 | ENSG00000277161 | 0.971480983 | miR-1285-y | 0.004842615 |
| ENST00000663483 | ENSG00000090776 | 0.9522661 | miR-1285-y | 0.02421308 |
| ENST00000663483 | ENSG00000107937 | 0.939615097 | miR-1285-y | 0.02421308 |
| ENST00000663483 | ENSG00000117525 | 0.980215464 | miR-1285-y | 0.02421308 |
| ENST00000663483 | ENSG00000173530 | 0.932317419 | miR-1285-y | 0.005085982 |
| MSTRG.10695.1 | ENSG00000090776 | 0.90634315 | miR-1285-y | 0.02421308 |
| MSTRG.10695.1 | ENSG00000105141 | 0.971108216 | miR-1285-y | 0.000100333 |
| MSTRG.10695.1 | ENSG00000117525 | 0.943808343 | miR-1285-y | 0.02421308 |
| MSTRG.10695.1 | ENSG00000136492 | 0.907121439 | miR-1285-y | 0.000100333 |
| MSTRG.10695.1 | ENSG00000145623 | 0.97150456 | miR-1285-y | 0.003091722 |
| MSTRG.10695.1 | ENSG00000148344 | 0.917550737 | miR-1285-y | 0.02421308 |
| MSTRG.12084.9 | ENSG00000090776 | 0.900530748 | miR-1285-y | 0.02663438 |
| MSTRG.13607.15 | ENSG00000085840 | 0.971835448 | miR-1285-y | 0.04358354 |
| MSTRG.13607.15 | ENSG00000092853 | 0.903428125 | miR-1285-y | 0.003411304 |
| MSTRG.13607.15 | ENSG00000148344 | 0.901789009 | miR-1285-y | 0.04358354 |
| MSTRG.13774.68 | ENSG00000117525 | 0.919443543 | miR-1285-y | 0.03874092 |
| MSTRG.13863.2 | ENSG00000136933 | 0.917834181 | miR-1285-y | 0.007263923 |
| MSTRG.13863.2 | ENSG00000198742 | 0.93292985 | miR-1285-y | 0.03596785 |
| MSTRG.18356.1 | ENSG00000090776 | 0.926388877 | miR-1285-y | 0.004842615 |
| MSTRG.18356.1 | ENSG00000105141 | 0.925802697 | miR-1285-y | 0.02409554 |
| MSTRG.18356.1 | ENSG00000107937 | 0.922932463 | miR-1285-y | 0.004842615 |
| MSTRG.18356.1 | ENSG00000117525 | 0.942683678 | miR-1285-y | 0.004842615 |
| MSTRG.18356.1 | ENSG00000173530 | 0.907727152 | miR-1285-y | 0.02409554 |
| MSTRG.18356.5 | ENSG00000105141 | 0.908088602 | miR-1285-y | 0.002047007 |
| MSTRG.18356.5 | ENSG00000136492 | 0.917076876 | miR-1285-y | 0.002047007 |
| MSTRG.18356.5 | ENSG00000145623 | 0.910309971 | miR-1285-y | 0.02117315 |
| MSTRG.18356.5 | ENSG00000165688 | 0.926910111 | miR-1285-y | 0.02117315 |
| MSTRG.18356.5 | ENSG00000173530 | 0.919007737 | miR-1285-y | 0.002047007 |
| MSTRG.18356.7 | ENSG00000165688 | 0.900040051 | miR-1285-y | 0.01262918 |
| MSTRG.19187.5 | ENSG00000085840 | 0.953229209 | miR-1285-y | 0.031477 |
| MSTRG.19187.5 | ENSG00000094804 | 0.962464835 | miR-1285-y | 0.002701343 |
| MSTRG.19187.5 | ENSG00000165688 | 0.958833623 | miR-1285-y | 0.005306331 |
| MSTRG.19187.5 | ENSG00000186854 | 0.900265935 | miR-1285-y | 0.031477 |
| MSTRG.1999.2 | ENSG00000090776 | 0.93306442 | miR-1285-y | 0.007263923 |
| MSTRG.1999.2 | ENSG00000105141 | 0.916750159 | miR-1285-y | 0.03596785 |
| MSTRG.1999.2 | ENSG00000107937 | 0.901597628 | miR-1285-y | 0.007263923 |
| MSTRG.1999.2 | ENSG00000117525 | 0.912229344 | miR-1285-y | 0.007263923 |
| MSTRG.1999.2 | ENSG00000145623 | 0.930574837 | miR-1285-y | 0.000210884 |
| MSTRG.1999.2 | ENSG00000160072 | 0.954863434 | miR-1285-y | 0.000210884 |
| MSTRG.21078.14 | ENSG00000090776 | 0.954894466 | miR-1285-y | 0.01694915 |
| MSTRG.21078.14 | ENSG00000107937 | 0.965665306 | miR-1285-y | 0.01694915 |
| MSTRG.21078.14 | ENSG00000117525 | 0.964303306 | miR-1285-y | 0.01694915 |
| MSTRG.2991.13 | ENSG00000101945 | 0.91923119 | miR-1285-y | 0.000441401 |
| MSTRG.6280.1 | ENSG00000117525 | 0.903635592 | miR-1285-y | 0.007263923 |
| MSTRG.6280.1 | ENSG00000165688 | 0.933242887 | miR-1285-y | 0.02884446 |
| MSTRG.6280.1 | ENSG00000186854 | 0.907433588 | miR-1285-y | 0.007263923 |
| MSTRG.6842.12 | ENSG00000090776 | 0.924159346 | miR-1285-y | 0.01452785 |
| MSTRG.6842.12 | ENSG00000107937 | 0.959347896 | miR-1285-y | 0.01452785 |
| MSTRG.6842.12 | ENSG00000117525 | 0.925365273 | miR-1285-y | 0.01452785 |
| MSTRG.6842.12 | ENSG00000145623 | 0.916114578 | miR-1285-y | 0.001044163 |
| MSTRG.6842.12 | ENSG00000160072 | 0.908479389 | miR-1285-y | 0.001044163 |
| MSTRG.8696.8 | ENSG00000085840 | 0.90334595 | miR-1285-y | 0.02905569 |
| MSTRG.8696.8 | ENSG00000090776 | 0.917990429 | miR-1285-y | 0.02905569 |
| MSTRG.8696.8 | ENSG00000094804 | 0.949242583 | miR-1285-y | 0.002289526 |
| MSTRG.8696.8 | ENSG00000117525 | 0.981980219 | miR-1285-y | 0.02905569 |
| MSTRG.8696.8 | ENSG00000145623 | 0.967108428 | miR-1285-y | 0.004504796 |
| MSTRG.8696.8 | ENSG00000148344 | 0.954919707 | miR-1285-y | 0.02905569 |
| MSTRG.8696.8 | ENSG00000165688 | 0.939413571 | miR-1285-y | 7.42567E-05 |
| MSTRG.8755.1 | ENSG00000146858 | 0.916754969 | miR-1285-y | 0.002421308 |
| MSTRG.8755.1 | ENSG00000173530 | 0.922182698 | miR-1285-y | 0.01210654 |
| ENST00000414532 | ENSG00000006432 | 0.925526386 | miR-1343-y | 0.02884446 |
| ENST00000414532 | ENSG00000106366 | 0.907611406 | miR-1343-y | 0.04305632 |
| ENST00000414532 | ENSG00000162377 | 0.919246109 | miR-1343-y | 0.04305632 |
| ENST00000414532 | ENSG00000170191 | 0.907512579 | miR-1343-y | 0.02884446 |
| ENST00000658077 | ENSG00000172175 | 0.957206821 | miR-1343-y | 1.7159E-06 |
| MSTRG.21558.5 | ENSG00000112658 | 0.901240485 | miR-1343-y | 0.000698384 |
| MSTRG.21558.5 | ENSG00000112715 | 0.906159829 | miR-1343-y | 0.04772434 |
| MSTRG.21558.5 | ENSG00000163814 | 0.918989136 | miR-1343-y | 0.003195884 |
| ENST00000366860 | ENSG00000124882 | 0.901444175 | miR-135-x | 0.02409554 |
| ENST00000491548 | ENSG00000134757 | 0.920062061 | miR-135-x | 0.00968523 |
| ENST00000535077 | ENSG00000105141 | 0.933604446 | miR-135-x | 0.01210654 |
| ENST00000535077 | ENSG00000124882 | 0.933722724 | miR-135-x | 0.004842615 |
| ENST00000643321 | ENSG00000105141 | 0.952344848 | miR-135-x | 0.01210654 |
| ENST00000643321 | ENSG00000124882 | 0.926701028 | miR-135-x | 0.004842615 |
| ENST00000643321 | ENSG00000162433 | 0.963981616 | miR-135-x | 0.004842615 |
| ENST00000643321 | ENSG00000171793 | 0.94892709 | miR-135-x | 0.004842615 |
| ENST00000643321 | ENSG00000173530 | 0.950347319 | miR-135-x | 0.01210654 |
| ENST00000657628 | ENSG00000082153 | 0.952965024 | miR-135-x | 0.01449258 |
| ENST00000657628 | ENSG00000087995 | 0.955151642 | miR-135-x | 0.004842615 |
| MSTRG.10695.1 | ENSG00000105141 | 0.971108216 | miR-135-x | 0.000100333 |
| MSTRG.10695.1 | ENSG00000124882 | 0.971222921 | miR-135-x | 0.000528926 |
| MSTRG.10695.1 | ENSG00000162433 | 0.921813434 | miR-135-x | 0.04789722 |
| MSTRG.10695.1 | ENSG00000171793 | 0.907989881 | miR-135-x | 0.04789722 |
| MSTRG.13952.1 | ENSG00000105141 | 0.926575095 | miR-135-x | 0.03596785 |
| MSTRG.13952.1 | ENSG00000124882 | 0.931637454 | miR-135-x | 0.01449258 |
| MSTRG.13952.1 | ENSG00000197971 | 0.935493942 | miR-135-x | 0.04305632 |
| MSTRG.18356.5 | ENSG00000105141 | 0.908088602 | miR-135-x | 0.002047007 |
| MSTRG.18356.5 | ENSG00000173530 | 0.919007737 | miR-135-x | 0.002047007 |
| ENST00000444059 | ENSG00000135074 | 0.912319554 | miR-3176-z | 0.02409554 |
| ENST00000444059 | ENSG00000189410 | 0.95684566 | miR-3176-z | 0.009673476 |
| ENST00000488648 | ENSG00000169067 | 0.907147851 | miR-3176-z | 0.02168607 |
| ENST00000488648 | ENSG00000177374 | 0.910122096 | miR-3176-z | 0.03596785 |
| ENST00000460490 | ENSG00000138316 | 0.932282688 | miR-324-x | 0.009673476 |
| ENST00000460490 | ENSG00000161960 | 0.904034296 | miR-324-x | 0.004842615 |
| ENST00000460490 | ENSG00000284934 | 0.984881689 | miR-324-x | 0.004842615 |
| ENST00000467894 | ENSG00000172175 | 0.919138043 | miR-324-x | 0.01452785 |
| MSTRG.24299.1 | ENSG00000197971 | 0.934503578 | miR-501-y | 9.34744E-05 |
| ENST00000411546 | ENSG00000162377 | 0.952333529 | miR-671-x | 6.82594E-06 |
| ENST00000411546 | ENSG00000163814 | 0.964519863 | miR-671-x | 1.90423E-05 |
| ENST00000397112 | ENSG00000189410 | 0.998099406 | novel-m0094-5p | 0.004842615 |
| ENST00000468817 | ENSG00000162377 | 0.927380858 | novel-m0094-5p | 0.02887938 |
| ENST00000468817 | ENSG00000163347 | 0.935010935 | novel-m0094-5p | 0.000117539 |
| ENST00000468817 | ENSG00000166197 | 0.933036602 | novel-m0094-5p | 0.009673476 |
| ENST00000468817 | ENSG00000197971 | 0.907593446 | novel-m0094-5p | 0.02887938 |
| ENST00000476682 | ENSG00000142627 | 0.948568075 | novel-m0094-5p | 0.02884446 |
| ENST00000476682 | ENSG00000158125 | 0.908032108 | novel-m0094-5p | 0.01929994 |
| ENST00000476682 | ENSG00000163347 | 0.93149094 | novel-m0094-5p | 0.04772434 |
| ENST00000476682 | ENSG00000198142 | 0.91657003 | novel-m0094-5p | 0.00968523 |
| ENST00000555112 | ENSG00000112658 | 0.92666804 | novel-m0094-5p | 0.01929994 |
| ENST00000555112 | ENSG00000142627 | 0.921535357 | novel-m0094-5p | 0.01449258 |
| ENST00000663439 | ENSG00000006432 | 0.929795043 | novel-m0094-5p | 0.00968523 |
| MSTRG.19604.1 | ENSG00000142627 | 0.939305797 | novel-m0094-5p | 0.04305632 |
| MSTRG.19604.1 | ENSG00000158125 | 0.925016576 | novel-m0094-5p | 0.000176309 |
| MSTRG.2060.1 | ENSG00000142627 | 0.904147201 | novel-m0094-5p | 0.02884446 |
| ENST00000427824 | ENSG00000004455 | 0.921425133 | novel-m0210-3p | 0.01210654 |
| ENST00000474026 | ENSG00000069399 | 0.957032608 | novel-m0210-3p | 0.02409554 |
| ENST00000658077 | ENSG00000172175 | 0.957206821 | novel-m0210-3p | 1.7159E-06 |
| ENST00000411546 | ENSG00000112715 | 0.91893582 | novel-m0297-5p | 0.000420398 |
| ENST00000411546 | ENSG00000117597 | 0.911887487 | novel-m0297-5p | 0.000698384 |
| ENST00000411546 | ENSG00000162377 | 0.952333529 | novel-m0297-5p | 6.82594E-06 |
| ENST00000411546 | ENSG00000163814 | 0.964519863 | novel-m0297-5p | 1.90423E-05 |
| ENST00000411546 | ENSG00000164543 | 0.937422456 | novel-m0297-5p | 0.000420398 |
| ENST00000536119 | ENSG00000169991 | 0.917848429 | novel-m0297-5p | 0.000350902 |
| ENST00000625013 | ENSG00000112715 | 0.926975941 | novel-m0297-5p | 0.000420398 |
| ENST00000625013 | ENSG00000162377 | 0.931462515 | novel-m0297-5p | 0.001044163 |
| ENST00000675579 | ENSG00000164543 | 0.900130423 | novel-m0297-5p | 0.000210884 |
| MSTRG.1296.4 | ENSG00000259330 | 0.951673834 | novel-m0297-5p | 0.000210884 |
| MSTRG.22911.2 | ENSG00000162377 | 0.905534496 | novel-m0297-5p | 0.002576559 |
| MSTRG.5477.4 | ENSG00000105928 | 0.943151773 | novel-m0297-5p | 0.001044163 |
| MSTRG.6417.1 | ENSG00000073756 | 0.926728861 | novel-m0297-5p | 0.003583685 |
| MSTRG.6417.1 | ENSG00000112715 | 0.93958329 | novel-m0297-5p | 0.001457059 |
| MSTRG.6417.1 | ENSG00000117597 | 0.913784634 | novel-m0297-5p | 0.002408705 |
| MSTRG.6417.1 | ENSG00000162377 | 0.977841219 | novel-m0297-5p | 0.003583685 |
| MSTRG.6417.1 | ENSG00000163814 | 0.929875989 | novel-m0297-5p | 0.000162078 |
| MSTRG.6417.1 | ENSG00000164543 | 0.917434617 | novel-m0297-5p | 0.001457059 |
| MSTRG.67.1 | ENSG00000148734 | 0.928833953 | miR-1247-x | 0.001728958 |
| ENST00000555985 | ENSG00000146197 | 0.975523399 | miR-1260-z | 0.008392455 |
| ENST00000555985 | ENSG00000185070 | 0.941390555 | miR-1260-z | 0.02968927 |
| MSTRG.13540.3 | ENSG00000177076 | 0.902600727 | miR-1260-z | 0.001598533 |
| MSTRG.13962.1 | ENSG00000145743 | 0.919714231 | miR-1260-z | 0.004181783 |
| MSTRG.13962.1 | ENSG00000146197 | 0.90748586 | miR-1260-z | 1.17431E-05 |
| MSTRG.13962.1 | ENSG00000163590 | 0.969635621 | miR-1260-z | 8.34953E-08 |
| MSTRG.13962.1 | ENSG00000185070 | 0.92571085 | miR-1260-z | 3.4704E-08 |
| MSTRG.14011.1 | ENSG00000146197 | 0.912229832 | miR-1260-z | 0.01106265 |
| MSTRG.14011.1 | ENSG00000150893 | 0.927133474 | miR-1260-z | 0.03841181 |
| MSTRG.14011.1 | ENSG00000165572 | 0.930016102 | miR-1260-z | 0.004747088 |
| MSTRG.14011.1 | ENSG00000177076 | 0.972035911 | miR-1260-z | 0.00032911 |
| MSTRG.14011.1 | ENSG00000181634 | 0.944046233 | miR-1260-z | 0.03841181 |
| MSTRG.14011.1 | ENSG00000185070 | 0.905808732 | miR-1260-z | 0.0386292 |
| MSTRG.1785.2 | ENSG00000145743 | 0.954497077 | miR-1260-z | 0.01316647 |
| MSTRG.1785.2 | ENSG00000146197 | 0.971162385 | miR-1260-z | 0.000170411 |
| MSTRG.1785.2 | ENSG00000185070 | 0.966291818 | miR-1260-z | 0.000210111 |
| MSTRG.22527.1 | ENSG00000150893 | 0.90436661 | miR-1260-z | 0.03841181 |
| MSTRG.22527.1 | ENSG00000163590 | 0.941712978 | miR-1260-z | 0.008689143 |
| MSTRG.22527.1 | ENSG00000177076 | 0.911232667 | miR-1260-z | 0.00032911 |
| MSTRG.24442.34 | ENSG00000145743 | 0.908648345 | miR-1260-z | 0.004568791 |
| MSTRG.24442.34 | ENSG00000146197 | 0.912110347 | miR-1260-z | 0.003616286 |
| MSTRG.2684.1 | ENSG00000150893 | 0.906763693 | miR-1260-z | 0.00179835 |
| MSTRG.6412.2 | ENSG00000165572 | 0.901832499 | miR-1260-z | 0.01089984 |
| MSTRG.67.1 | ENSG00000148734 | 0.928833953 | miR-1247-x | 0.001728958 |
| MSTRG.67.1 | ENSG00000177076 | 0.940758277 | miR-1260-z | 0.02887938 |
| MSTRG.67.1 | ENSG00000181634 | 0.939180101 | miR-1260-z | 0.02887938 |
| ENST00000454129 | ENSG00000213463 | 0.936495964 | miR-3963-z | 0.03831915 |
| ENST00000463567 | ENSG00000169242 | 0.90402755 | miR-3963-z | 0.001566188 |
| ENST00000463567 | ENSG00000176438 | 0.929417346 | miR-3963-z | 0.002015928 |
| ENST00000463567 | ENSG00000265491 | 0.932105035 | miR-3963-z | 0.02143896 |
| ENST00000478190 | ENSG00000146197 | 0.969618147 | miR-3963-z | 0.00232167 |
| ENST00000478190 | ENSG00000185070 | 0.948711285 | miR-3963-z | 0.01596083 |
| ENST00000478190 | ENSG00000185352 | 0.906935729 | miR-3963-z | 0.02036195 |
| ENST00000503611 | ENSG00000186026 | 0.933790825 | miR-3963-z | 0.03596785 |
| ENST00000505532 | ENSG00000011201 | 0.938954304 | miR-3963-z | 0.000525495 |
| ENST00000505532 | ENSG00000064787 | 0.930897004 | miR-3963-z | 0.00606356 |
| ENST00000505532 | ENSG00000131171 | 0.951100167 | miR-3963-z | 0.001044163 |
| ENST00000505532 | ENSG00000185070 | 0.914951567 | miR-3963-z | 0.02173251 |
| ENST00000505532 | ENSG00000205269 | 0.920081082 | miR-3963-z | 0.004747088 |
| ENST00000635449 | ENSG00000152409 | 0.929060028 | miR-3963-z | 0.01525231 |
| ENST00000635449 | ENSG00000205269 | 0.92723444 | miR-3963-z | 0.006200617 |
| ENST00000646803 | ENSG00000011201 | 0.947884405 | miR-3963-z | 0.003624411 |
| ENST00000646803 | ENSG00000033122 | 0.905914089 | miR-3963-z | 0.002045656 |
| ENST00000646803 | ENSG00000064787 | 0.964932692 | miR-3963-z | 0.03828835 |
| ENST00000646803 | ENSG00000134202 | 0.931618737 | miR-3963-z | 0.003624411 |
| ENST00000646803 | ENSG00000146197 | 0.90651462 | miR-3963-z | 0.002869814 |
| ENST00000646803 | ENSG00000167653 | 0.903436264 | miR-3963-z | 0.001234162 |
| ENST00000646803 | ENSG00000168874 | 0.93727234 | miR-3963-z | 0.03828835 |
| ENST00000646803 | ENSG00000169242 | 0.912467212 | miR-3963-z | 0.003624411 |
| ENST00000646803 | ENSG00000172201 | 0.956102548 | miR-3963-z | 0.007096103 |
| ENST00000646803 | ENSG00000176438 | 0.962979697 | miR-3963-z | 0.00703067 |
| ENST00000646803 | ENSG00000185070 | 0.928700147 | miR-3963-z | 0.002045656 |
| ENST00000646803 | ENSG00000185352 | 0.961593326 | miR-3963-z | 0.02330223 |
| ENST00000646803 | ENSG00000205269 | 0.914513779 | miR-3963-z | 0.00195624 |
| ENST00000646803 | ENSG00000265491 | 0.902038869 | miR-3963-z | 0.04685806 |
| ENST00000659656 | ENSG00000033122 | 0.921492569 | miR-3963-z | 0.005837757 |
| ENST00000659656 | ENSG00000164938 | 0.935064257 | miR-3963-z | 0.001128543 |
| ENST00000659656 | ENSG00000168874 | 0.931638914 | miR-3963-z | 0.01737688 |
| ENST00000659656 | ENSG00000265491 | 0.914732967 | miR-3963-z | 0.02143896 |
| ENST00000659936 | ENSG00000035664 | 0.950770672 | miR-3963-z | 0.04685806 |
| ENST00000659936 | ENSG00000123407 | 0.941916842 | miR-3963-z | 0.003091722 |
| ENST00000659936 | ENSG00000146197 | 0.919969079 | miR-3963-z | 0.01737688 |
| ENST00000659936 | ENSG00000163430 | 0.902476367 | miR-3963-z | 0.003091722 |
| ENST00000659936 | ENSG00000164938 | 0.917033995 | miR-3963-z | 0.02143896 |
| ENST00000659936 | ENSG00000168874 | 0.901139364 | miR-3963-z | 0.01737688 |
| ENST00000659936 | ENSG00000172201 | 0.907807697 | miR-3963-z | 0.003091722 |
| ENST00000659936 | ENSG00000176438 | 0.944279125 | miR-3963-z | 0.03063217 |
| ENST00000659936 | ENSG00000185352 | 0.957557325 | miR-3963-z | 0.000342206 |
| ENST00000659936 | ENSG00000265491 | 0.905601439 | miR-3963-z | 0.001128543 |
| ENST00000666841 | ENSG00000168874 | 0.926990837 | miR-3963-z | 0.02099658 |
| ENST00000666841 | ENSG00000172201 | 0.915892906 | miR-3963-z | 0.003766373 |
| ENST00000675700 | ENSG00000131171 | 0.931938969 | miR-3963-z | 0.006170319 |
| ENST00000675700 | ENSG00000148734 | 0.937651654 | miR-3963-z | 0.01008397 |
| ENST00000675700 | ENSG00000152409 | 0.932941158 | miR-3963-z | 0.006170319 |
| ENST00000675700 | ENSG00000185070 | 0.951128482 | miR-3963-z | 0.01596083 |
| ENST00000675700 | ENSG00000205269 | 0.908767069 | miR-3963-z | 0.02662272 |
| MSTRG.10951.4 | ENSG00000033122 | 0.936341939 | miR-3963-z | 0.007821167 |
| MSTRG.10951.4 | ENSG00000100234 | 0.913487117 | miR-3963-z | 0.000646466 |
| MSTRG.10951.4 | ENSG00000134202 | 0.945534576 | miR-3963-z | 0.001911084 |
| MSTRG.10951.4 | ENSG00000164938 | 0.978200483 | miR-3963-z | 0.001531728 |
| MSTRG.10951.4 | ENSG00000168874 | 0.94727186 | miR-3963-z | 0.02099658 |
| MSTRG.10951.4 | ENSG00000169242 | 0.927988042 | miR-3963-z | 0.001911084 |
| MSTRG.10951.4 | ENSG00000172201 | 0.921673489 | miR-3963-z | 0.003766373 |
| MSTRG.10951.4 | ENSG00000176438 | 0.9350837 | miR-3963-z | 0.03683353 |
| MSTRG.10951.4 | ENSG00000185352 | 0.916361988 | miR-3963-z | 0.01261345 |
| MSTRG.10951.4 | ENSG00000265491 | 0.925378043 | miR-3963-z | 0.02586279 |
| MSTRG.13249.1 | ENSG00000185352 | 0.946160419 | miR-3963-z | 0.008392455 |
| MSTRG.13540.2 | ENSG00000152409 | 0.949898444 | miR-3963-z | 0.000272351 |
| MSTRG.13540.2 | ENSG00000205269 | 0.915457208 | miR-3963-z | 0.04303143 |
| MSTRG.13962.1 | ENSG00000146197 | 0.90748586 | miR-3963-z | 1.17431E-05 |
| MSTRG.13962.1 | ENSG00000152409 | 0.933305774 | miR-3963-z | 3.42511E-05 |
| MSTRG.13962.1 | ENSG00000185070 | 0.92571085 | miR-3963-z | 3.4704E-08 |
| MSTRG.13962.1 | ENSG00000205269 | 0.913207451 | miR-3963-z | 4.1508E-06 |
| MSTRG.18465.1 | ENSG00000053254 | 0.929204379 | miR-3963-z | 0.009143053 |
| MSTRG.18465.1 | ENSG00000100234 | 0.915263959 | miR-3963-z | 0.000646466 |
| MSTRG.18465.1 | ENSG00000134202 | 0.903919766 | miR-3963-z | 0.001911084 |
| MSTRG.18465.1 | ENSG00000168874 | 0.942449194 | miR-3963-z | 3.24786E-05 |
| MSTRG.18465.1 | ENSG00000169242 | 0.94290436 | miR-3963-z | 0.001911084 |
| MSTRG.18465.1 | ENSG00000172201 | 0.944919334 | miR-3963-z | 5.57961E-05 |
| MSTRG.18465.1 | ENSG00000221909 | 0.918734248 | miR-3963-z | 0.02663438 |
| MSTRG.19535.1 | ENSG00000265491 | 0.908325889 | miR-3963-z | 0.01777699 |
| MSTRG.20269.1 | ENSG00000011201 | 0.908915233 | miR-3963-z | 0.000350902 |
| MSTRG.20269.1 | ENSG00000033122 | 0.921560802 | miR-3963-z | 0.000553904 |
| MSTRG.20269.1 | ENSG00000064787 | 0.950671232 | miR-3963-z | 7.04936E-05 |
| MSTRG.20269.1 | ENSG00000134202 | 0.939109819 | miR-3963-z | 8.5795E-07 |
| MSTRG.20269.1 | ENSG00000146197 | 0.911655903 | miR-3963-z | 0.004088852 |
| MSTRG.20269.1 | ENSG00000168874 | 0.923103205 | miR-3963-z | 0.004088852 |
| MSTRG.20269.1 | ENSG00000169242 | 0.904167111 | miR-3963-z | 0.000350902 |
| MSTRG.20269.1 | ENSG00000172201 | 0.942648768 | miR-3963-z | 0.000698384 |
| MSTRG.20269.1 | ENSG00000176438 | 0.900590498 | miR-3963-z | 0.000182583 |
| MSTRG.20269.1 | ENSG00000185070 | 0.930987888 | miR-3963-z | 0.000553904 |
| MSTRG.20269.1 | ENSG00000185352 | 0.936444172 | miR-3963-z | 0.002408705 |
| MSTRG.20889.1 | ENSG00000033122 | 0.935919858 | miR-3963-z | 0.002045656 |
| MSTRG.20889.1 | ENSG00000100234 | 0.916833842 | miR-3963-z | 0.001234162 |
| MSTRG.20889.1 | ENSG00000134202 | 0.964416028 | miR-3963-z | 0.003624411 |
| MSTRG.20889.1 | ENSG00000164938 | 0.952812922 | miR-3963-z | 6.98222E-06 |
| MSTRG.20889.1 | ENSG00000168874 | 0.960001918 | miR-3963-z | 0.002869814 |
| MSTRG.20889.1 | ENSG00000169242 | 0.930215594 | miR-3963-z | 0.003624411 |
| MSTRG.20889.1 | ENSG00000172201 | 0.947536867 | miR-3963-z | 0.007096103 |
| MSTRG.20889.1 | ENSG00000185352 | 0.91612404 | miR-3963-z | 0.02330223 |
| MSTRG.20889.1 | ENSG00000265491 | 0.942956899 | miR-3963-z | 0.000210501 |
| MSTRG.20957.1 | ENSG00000033122 | 0.965361694 | miR-3963-z | 0.008462146 |
| MSTRG.20957.1 | ENSG00000064787 | 0.953403808 | miR-3963-z | 0.03044213 |
| MSTRG.20957.1 | ENSG00000100234 | 0.924135909 | miR-3963-z | 0.006593949 |
| MSTRG.20957.1 | ENSG00000134202 | 0.964942026 | miR-3963-z | 0.000513398 |
| MSTRG.20957.1 | ENSG00000164938 | 0.961489249 | miR-3963-z | 0.005674705 |
| MSTRG.20957.1 | ENSG00000168874 | 0.985202263 | miR-3963-z | 0.03044213 |
| MSTRG.20957.1 | ENSG00000169242 | 0.967271837 | miR-3963-z | 0.01875505 |
| MSTRG.20957.1 | ENSG00000172201 | 0.96946002 | miR-3963-z | 0.03557297 |
| MSTRG.20957.1 | ENSG00000185070 | 0.906219357 | miR-3963-z | 0.04225439 |
| MSTRG.20957.1 | ENSG00000185352 | 0.921697987 | miR-3963-z | 0.01423784 |
| MSTRG.20957.1 | ENSG00000265491 | 0.952119664 | miR-3963-z | 0.000528659 |
| MSTRG.21997.7 | ENSG00000146197 | 0.90008518 | miR-3963-z | 0.00232167 |
| MSTRG.21997.7 | ENSG00000185070 | 0.920816484 | miR-3963-z | 0.001539649 |
| MSTRG.22049.1 | ENSG00000033122 | 0.992000178 | miR-3963-z | 0.02173251 |
| MSTRG.22049.1 | ENSG00000064787 | 0.943960471 | miR-3963-z | 0.00606356 |
| MSTRG.22049.1 | ENSG00000100234 | 0.96939884 | miR-3963-z | 0.000176309 |
| MSTRG.22049.1 | ENSG00000134202 | 0.988444666 | miR-3963-z | 0.000525495 |
| MSTRG.22049.1 | ENSG00000164938 | 0.940768238 | miR-3963-z | 0.007529928 |
| MSTRG.22049.1 | ENSG00000168874 | 0.967911468 | miR-3963-z | 0.00606356 |
| MSTRG.22049.1 | ENSG00000169242 | 0.953121466 | miR-3963-z | 0.000525495 |
| MSTRG.22049.1 | ENSG00000172201 | 0.95167218 | miR-3963-z | 0.001044163 |
| MSTRG.22049.1 | ENSG00000176438 | 0.928214444 | miR-3963-z | 0.01089984 |
| MSTRG.22049.1 | ENSG00000265491 | 0.940601804 | miR-3963-z | 0.007529928 |
| MSTRG.22809.1 | ENSG00000146197 | 0.982227603 | miR-3963-z | 0.008392455 |
| MSTRG.22809.1 | ENSG00000167653 | 0.90164585 | miR-3963-z | 0.03365147 |
| MSTRG.22809.1 | ENSG00000168874 | 0.937799452 | miR-3963-z | 0.008392455 |
| MSTRG.22809.1 | ENSG00000185070 | 0.950652108 | miR-3963-z | 0.02968927 |
| MSTRG.22809.1 | ENSG00000265491 | 0.926704335 | miR-3963-z | 0.01040502 |
| MSTRG.23877.1 | ENSG00000164938 | 0.925270496 | miR-3963-z | 0.01737688 |
| MSTRG.23877.1 | ENSG00000185070 | 0.902683641 | miR-3963-z | 0.04846785 |
| MSTRG.2430.1 | ENSG00000033122 | 0.907620959 | miR-3963-z | 0.03731622 |
| MSTRG.2430.1 | ENSG00000186026 | 0.90736071 | miR-3963-z | 0.00586349 |
| MSTRG.2430.1 | ENSG00000205269 | 0.908227953 | miR-3963-z | 0.04762182 |
| MSTRG.24442.34 | ENSG00000146197 | 0.912110347 | miR-3963-z | 0.003616286 |
| MSTRG.2672.1 | ENSG00000035664 | 0.956152136 | miR-3963-z | 0.04685806 |
| MSTRG.2672.1 | ENSG00000134202 | 0.912916117 | miR-3963-z | 0.001566188 |
| MSTRG.2672.1 | ENSG00000164938 | 0.905300651 | miR-3963-z | 0.02143896 |
| MSTRG.2672.1 | ENSG00000167653 | 0.909519296 | miR-3963-z | 0.04789722 |
| MSTRG.2672.1 | ENSG00000176438 | 0.954123229 | miR-3963-z | 0.002015928 |
| MSTRG.2672.1 | ENSG00000265491 | 0.916844275 | miR-3963-z | 0.02143896 |
| MSTRG.2684.1 | ENSG00000185352 | 0.931966487 | miR-3963-z | 0.0331264 |
| MSTRG.4416.1 | ENSG00000146197 | 0.903328913 | miR-3963-z | 0.03044213 |
| MSTRG.4416.1 | ENSG00000152409 | 0.918497685 | miR-3963-z | 0.03557297 |
| MSTRG.4416.1 | ENSG00000185070 | 0.928301917 | miR-3963-z | 0.008462146 |
| MSTRG.4416.1 | ENSG00000205269 | 0.943037726 | miR-3963-z | 0.002133931 |
| MSTRG.6489.2 | ENSG00000185352 | 0.911542354 | miR-3963-z | 0.0331264 |
| MSTRG.685.2 | ENSG00000033122 | 0.952445736 | miR-3963-z | 0.03731622 |
| MSTRG.685.2 | ENSG00000100234 | 0.920913652 | miR-3963-z | 0.00200992 |
| MSTRG.685.2 | ENSG00000134202 | 0.959223445 | miR-3963-z | 8.31354E-05 |
| MSTRG.685.2 | ENSG00000164938 | 0.903512846 | miR-3963-z | 0.000569635 |
| MSTRG.685.2 | ENSG00000168874 | 0.929391407 | miR-3963-z | 0.005843617 |
| MSTRG.685.2 | ENSG00000169242 | 0.901669562 | miR-3963-z | 0.00586349 |
| MSTRG.685.2 | ENSG00000172201 | 0.908600098 | miR-3963-z | 0.01140417 |
| MSTRG.685.2 | ENSG00000265491 | 0.936715344 | miR-3963-z | 0.000569635 |
| ENST00000637997 | ENSG00000172817 | 0.934323939 | miR-4286-z | 0.04772434 |
| MSTRG.13962.1 | ENSG00000185070 | 0.92571085 | miR-4286-z | 3.4704E-08 |
| MSTRG.1785.2 | ENSG00000185070 | 0.966291818 | miR-4286-z | 0.000210111 |
| ENST00000427447 | ENSG00000176399 | 0.910124422 | miR-4455-z | 0.007096103 |
| ENST00000427447 | ENSG00000277443 | 0.912129963 | miR-4455-z | 0.03631961 |
| ENST00000498724 | ENSG00000088367 | 0.920503512 | miR-4455-z | 0.03841181 |
| ENST00000498724 | ENSG00000112902 | 0.969358875 | miR-4455-z | 0.03841181 |
| ENST00000498724 | ENSG00000277443 | 0.934319359 | miR-4455-z | 0.004842615 |
| ENST00000603037 | ENSG00000205269 | 0.921903383 | miR-4455-z | 0.003411304 |
| ENST00000603037 | ENSG00000277443 | 0.903014165 | miR-4455-z | 0.04358354 |
| ENST00000644721 | ENSG00000088367 | 0.951841907 | miR-4455-z | 0.004013128 |
| ENST00000644721 | ENSG00000101417 | 0.905897525 | miR-4455-z | 0.04762182 |
| ENST00000644721 | ENSG00000176399 | 0.957014506 | miR-4455-z | 0.01140417 |
| ENST00000644721 | ENSG00000277443 | 0.932407988 | miR-4455-z | 0.04600484 |
| ENST00000646803 | ENSG00000146197 | 0.90651462 | miR-4455-z | 0.002869814 |
| ENST00000646803 | ENSG00000155621 | 0.929146654 | miR-4455-z | 0.01700163 |
| ENST00000646803 | ENSG00000176399 | 0.984282037 | miR-4455-z | 0.007096103 |
| ENST00000646803 | ENSG00000185070 | 0.928700147 | miR-4455-z | 0.002045656 |
| ENST00000646803 | ENSG00000205269 | 0.914513779 | miR-4455-z | 0.00195624 |
| ENST00000646803 | ENSG00000277443 | 0.924965914 | miR-4455-z | 0.03631961 |
| ENST00000659656 | ENSG00000088367 | 0.917282416 | miR-4455-z | 0.01369319 |
| ENST00000659656 | ENSG00000101417 | 0.933352865 | miR-4455-z | 0.000540488 |
| ENST00000659656 | ENSG00000110975 | 0.931503961 | miR-4455-z | 0.003091722 |
| MSTRG.13961.14 | ENSG00000138336 | 0.904844423 | miR-4455-z | 9.78063E-05 |
| MSTRG.13961.14 | ENSG00000163590 | 0.919734868 | miR-4455-z | 0.00467722 |
| MSTRG.13961.14 | ENSG00000169967 | 0.923365813 | miR-4455-z | 1.96423E-05 |
| MSTRG.13961.14 | ENSG00000185070 | 0.945377687 | miR-4455-z | 0.006374031 |
| MSTRG.13961.14 | ENSG00000205269 | 0.912529859 | miR-4455-z | 0.00467722 |
| MSTRG.13962.1 | ENSG00000146197 | 0.90748586 | miR-4455-z | 1.17431E-05 |
| MSTRG.13962.1 | ENSG00000163590 | 0.969635621 | miR-4455-z | 8.34953E-08 |
| MSTRG.13962.1 | ENSG00000169967 | 0.936316192 | miR-4455-z | 0.000161541 |
| MSTRG.13962.1 | ENSG00000185070 | 0.92571085 | miR-4455-z | 3.4704E-08 |
| MSTRG.13962.1 | ENSG00000205269 | 0.913207451 | miR-4455-z | 4.1508E-06 |
| MSTRG.15127.1 | ENSG00000176399 | 0.965973823 | miR-4455-z | 0.01023771 |
| MSTRG.19632.1 | ENSG00000176399 | 0.96717369 | miR-4455-z | 0.005306331 |
| MSTRG.19632.1 | ENSG00000277443 | 0.902244985 | miR-4455-z | 0.031477 |
| MSTRG.21997.7 | ENSG00000146197 | 0.90008518 | miR-4455-z | 0.00232167 |
| MSTRG.21997.7 | ENSG00000185070 | 0.920816484 | miR-4455-z | 0.001539649 |
| MSTRG.2714.1 | ENSG00000088367 | 0.951471849 | miR-4455-z | 0.007997136 |
| MSTRG.2714.1 | ENSG00000101417 | 0.916642179 | miR-4455-z | 0.007997136 |
| MSTRG.2714.1 | ENSG00000110975 | 0.926246364 | miR-4455-z | 0.01810198 |
| MSTRG.2714.1 | ENSG00000112902 | 0.900105421 | miR-4455-z | 0.000530702 |
| MSTRG.2714.1 | ENSG00000176399 | 0.950010204 | miR-4455-z | 0.01810198 |
| MSTRG.2714.2 | ENSG00000138336 | 0.961111102 | miR-4455-z | 0.002701343 |
| MSTRG.2714.2 | ENSG00000146197 | 0.94644076 | miR-4455-z | 0.02910242 |
| MSTRG.2714.2 | ENSG00000169967 | 0.962133528 | miR-4455-z | 0.008686234 |
| MSTRG.2714.2 | ENSG00000185070 | 0.984084471 | miR-4455-z | 0.001128683 |
| MSTRG.2714.2 | ENSG00000205269 | 0.965704572 | miR-4455-z | 0.02304514 |
| MSTRG.4416.1 | ENSG00000138336 | 0.943830534 | miR-4455-z | 0.01875505 |
| MSTRG.4416.1 | ENSG00000146197 | 0.903328913 | miR-4455-z | 0.03044213 |
| MSTRG.4416.1 | ENSG00000169967 | 0.908036052 | miR-4455-z | 0.004568791 |
| MSTRG.4416.1 | ENSG00000185070 | 0.928301917 | miR-4455-z | 0.008462146 |
| MSTRG.4416.1 | ENSG00000205269 | 0.943037726 | miR-4455-z | 0.002133931 |
| MSTRG.685.2 | ENSG00000088367 | 0.903548075 | miR-4455-z | 0.04762182 |
| MSTRG.685.2 | ENSG00000110975 | 0.918972488 | miR-4455-z | 0.01140417 |
| MSTRG.933.1 | ENSG00000088367 | 0.942513782 | miR-4455-z | 0.01369319 |
| ENST00000555985 | ENSG00000146197 | 0.975523399 | miR-4695-y | 0.008392455 |
| ENST00000555985 | ENSG00000185070 | 0.941390555 | miR-4695-y | 0.02968927 |
| ENST00000566733 | ENSG00000101417 | 0.931334768 | miR-4695-y | 0.000989787 |
| ENST00000566733 | ENSG00000112378 | 0.953480453 | miR-4695-y | 0.000233502 |
| ENST00000566733 | ENSG00000145743 | 0.912672288 | miR-4695-y | 8.16678E-05 |
| ENST00000566733 | ENSG00000146197 | 0.939028524 | miR-4695-y | 0.01784551 |
| ENST00000566733 | ENSG00000168874 | 0.987726324 | miR-4695-y | 0.001698011 |
| ENST00000566733 | ENSG00000177694 | 0.96223109 | miR-4695-y | 0.000989787 |
| ENST00000566733 | ENSG00000185070 | 0.925244238 | miR-4695-y | 0.000415149 |
| MSTRG.13540.3 | ENSG00000177076 | 0.902600727 | miR-4695-y | 0.001598533 |
| MSTRG.13962.1 | ENSG00000145743 | 0.919714231 | miR-4695-y | 0.004181783 |
| MSTRG.13962.1 | ENSG00000146197 | 0.90748586 | miR-4695-y | 1.17431E-05 |
| MSTRG.13962.1 | ENSG00000163590 | 0.969635621 | miR-4695-y | 8.34953E-08 |
| MSTRG.13962.1 | ENSG00000185070 | 0.92571085 | miR-4695-y | 3.4704E-08 |
| MSTRG.14011.1 | ENSG00000146197 | 0.912229832 | miR-4695-y | 0.01106265 |
| MSTRG.14011.1 | ENSG00000177076 | 0.972035911 | miR-4695-y | 0.00032911 |
| MSTRG.14011.1 | ENSG00000185070 | 0.905808732 | miR-4695-y | 0.0386292 |
| MSTRG.1785.2 | ENSG00000101417 | 0.905348762 | miR-4695-y | 0.03442217 |
| MSTRG.1785.2 | ENSG00000145743 | 0.954497077 | miR-4695-y | 0.01316647 |
| MSTRG.1785.2 | ENSG00000146197 | 0.971162385 | miR-4695-y | 0.000170411 |
| MSTRG.1785.2 | ENSG00000185070 | 0.966291818 | miR-4695-y | 0.000210111 |
| MSTRG.18465.1 | ENSG00000053254 | 0.929204379 | miR-4695-y | 0.009143053 |
| MSTRG.18465.1 | ENSG00000088367 | 0.905604698 | miR-4695-y | 0.0165725 |
| MSTRG.18465.1 | ENSG00000112378 | 0.959814799 | miR-4695-y | 0.009143053 |
| MSTRG.18465.1 | ENSG00000168874 | 0.942449194 | miR-4695-y | 3.24786E-05 |
| MSTRG.21233.1 | ENSG00000161714 | 0.959703147 | miR-4695-y | 0.003583685 |
| MSTRG.22527.1 | ENSG00000163590 | 0.941712978 | miR-4695-y | 0.008689143 |
| MSTRG.22527.1 | ENSG00000177076 | 0.911232667 | miR-4695-y | 0.00032911 |
| MSTRG.24442.34 | ENSG00000145743 | 0.908648345 | miR-4695-y | 0.004568791 |
| MSTRG.24442.34 | ENSG00000146197 | 0.912110347 | miR-4695-y | 0.003616286 |
| ENST00000478190 | ENSG00000143217 | 0.901544695 | novel-m0013-5p | 0.03389831 |
| ENST00000478190 | ENSG00000146197 | 0.969618147 | novel-m0013-5p | 0.00232167 |
| ENST00000478190 | ENSG00000184731 | 0.906017924 | novel-m0013-5p | 0.01008397 |
| ENST00000478190 | ENSG00000185070 | 0.948711285 | novel-m0013-5p | 0.01596083 |
| ENST00000556691 | ENSG00000106031 | 0.958595425 | novel-m0013-5p | 0.002421308 |
| ENST00000562691 | ENSG00000104154 | 0.960967735 | novel-m0013-5p | 3.74601E-05 |
| ENST00000562691 | ENSG00000112079 | 0.944827294 | novel-m0013-5p | 0.0115779 |
| ENST00000562691 | ENSG00000126803 | 0.962668842 | novel-m0013-5p | 0.003624411 |
| ENST00000562691 | ENSG00000146197 | 0.952524484 | novel-m0013-5p | 0.03828835 |
| ENST00000562691 | ENSG00000150907 | 0.930254646 | novel-m0013-5p | 0.02330223 |
| ENST00000562691 | ENSG00000168874 | 0.919425697 | novel-m0013-5p | 0.002869814 |
| ENST00000562691 | ENSG00000177694 | 0.908503086 | novel-m0013-5p | 0.03041756 |
| ENST00000562691 | ENSG00000184731 | 0.953524011 | novel-m0013-5p | 0.0115779 |
| ENST00000562691 | ENSG00000185070 | 0.962424877 | novel-m0013-5p | 0.002045656 |
| ENST00000571197 | ENSG00000106031 | 0.951074983 | novel-m0013-5p | 0.007263923 |
| ENST00000571197 | ENSG00000126803 | 0.93103843 | novel-m0013-5p | 0.02168607 |
| ENST00000587317 | ENSG00000172817 | 0.933063056 | novel-m0013-5p | 0.00968523 |
| ENST00000608395 | ENSG00000184731 | 0.921294331 | novel-m0013-5p | 0.01662139 |
| ENST00000608395 | ENSG00000205269 | 0.934053235 | novel-m0013-5p | 0.003411304 |
| MSTRG.13962.1 | ENSG00000146197 | 0.90748586 | novel-m0013-5p | 1.17431E-05 |
| MSTRG.13962.1 | ENSG00000169967 | 0.936316192 | novel-m0013-5p | 0.000161541 |
| MSTRG.13962.1 | ENSG00000185070 | 0.92571085 | novel-m0013-5p | 3.4704E-08 |
| MSTRG.13962.1 | ENSG00000205269 | 0.913207451 | novel-m0013-5p | 4.1508E-06 |
| MSTRG.1785.2 | ENSG00000101417 | 0.905348762 | novel-m0013-5p | 0.03442217 |
| MSTRG.1785.2 | ENSG00000146197 | 0.971162385 | novel-m0013-5p | 0.000170411 |
| MSTRG.1785.2 | ENSG00000185070 | 0.966291818 | novel-m0013-5p | 0.000210111 |
| MSTRG.1785.2 | ENSG00000205269 | 0.919339427 | novel-m0013-5p | 0.03442217 |
| MSTRG.19535.1 | ENSG00000112079 | 0.933287298 | novel-m0013-5p | 0.03147184 |
| MSTRG.19535.1 | ENSG00000265491 | 0.908325889 | novel-m0013-5p | 0.01777699 |
| MSTRG.2141.1 | ENSG00000088367 | 0.948756756 | novel-m0013-5p | 0.000201572 |
| MSTRG.2141.1 | ENSG00000101417 | 0.91498554 | novel-m0013-5p | 0.000201572 |
| MSTRG.2141.1 | ENSG00000104154 | 0.992041513 | novel-m0013-5p | 0.002583795 |
| MSTRG.2141.1 | ENSG00000146197 | 0.927492362 | novel-m0013-5p | 0.000352152 |
| MSTRG.2141.1 | ENSG00000164938 | 0.941205438 | novel-m0013-5p | 0.000569635 |
| MSTRG.2141.1 | ENSG00000167767 | 0.948589428 | novel-m0013-5p | 0.01848468 |
| MSTRG.2141.1 | ENSG00000168874 | 0.988987472 | novel-m0013-5p | 0.005843617 |
| MSTRG.2141.1 | ENSG00000169967 | 0.92338226 | novel-m0013-5p | 0.01848468 |
| MSTRG.2141.1 | ENSG00000177694 | 0.965393375 | novel-m0013-5p | 0.04762182 |
| MSTRG.2141.1 | ENSG00000184731 | 0.985850376 | novel-m0013-5p | 0.01848468 |
| MSTRG.2141.1 | ENSG00000185070 | 0.935896358 | novel-m0013-5p | 0.005234055 |
| MSTRG.2141.1 | ENSG00000205269 | 0.925521797 | novel-m0013-5p | 0.04762182 |
| MSTRG.2141.1 | ENSG00000265491 | 0.949148683 | novel-m0013-5p | 0.000569635 |
| MSTRG.22809.1 | ENSG00000146197 | 0.982227603 | novel-m0013-5p | 0.008392455 |
| MSTRG.22809.1 | ENSG00000168874 | 0.937799452 | novel-m0013-5p | 0.008392455 |
| MSTRG.22809.1 | ENSG00000185070 | 0.950652108 | novel-m0013-5p | 0.02968927 |
| MSTRG.22809.1 | ENSG00000265491 | 0.926704335 | novel-m0013-5p | 0.01040502 |
| MSTRG.685.2 | ENSG00000088367 | 0.903548075 | novel-m0013-5p | 0.04762182 |
| MSTRG.685.2 | ENSG00000164938 | 0.903512846 | novel-m0013-5p | 0.000569635 |
| MSTRG.685.2 | ENSG00000168874 | 0.929391407 | novel-m0013-5p | 0.005843617 |
| MSTRG.685.2 | ENSG00000177694 | 0.947826532 | novel-m0013-5p | 0.04762182 |
| MSTRG.685.2 | ENSG00000265491 | 0.936715344 | novel-m0013-5p | 0.000569635 |
| MSTRG.13988.1 | ENSG00000142188 | 0.920267529 | novel-m0046-3p | 5.07672E-05 |
| MSTRG.13988.1 | ENSG00000171368 | 0.957725222 | novel-m0046-3p | 1.79084E-07 |
| ENST00000477495 | ENSG00000151233 | 0.913421818 | novel-m0139-5p | 0.000210884 |
| ENST00000477495 | ENSG00000165572 | 0.922889023 | novel-m0139-5p | 0.001044163 |
